# Identification of Predictors for Weight Reduction in Children and Adolescents with Overweight and Obesity (IDA-Insel Survey)

**DOI:** 10.3390/healthcare4010005

**Published:** 2016-01-07

**Authors:** Ralf Schiel, Alexander Kaps, Günter Stein, Antje Steveling

**Affiliations:** 1 MEDIGREIF-Inselklinik Heringsdorf GmbH, Fachklinik für Kinder und Jugendliche, D-17424 Heringsdorf, Germany; alexander_kaps@yahoo.de; 2 Mathias-Hochschule, University of Applied Sciences, D-48431 Rheine, Germany; r.schiel@mhrheine.de; 3 Internal Medicine, Friedrich-Schiller-University, D-07745 Jena, Germany; stein.guenter37@gmail.com; 4 Internal Medicine A, Ernst-Moritz-Arndt-University, D-17489 Greifswald, Germany; antje.steveling@uni-greifswald.de

**Keywords:** body-mass index, carotid intima-media thickness, quality of life, resilience

## Abstract

*Introduction*: Worldwide, overweight and obesity are known as posing serious health risks. Successful methods of prevention and therapy for overweight and obesity have remained elusive. It was the aim of the present trial to identify parameters and determinants to guarantee long-term weight reduction. *Patients and methods*: In total 143/159 children and adolescents (90%) with overweight and obesity completed the prospective, multicenter trial (age 13.9 ± 2.4 years, BMI 31.2 ± 5.4 kg/m^2^, BMI-SDS 2.51 ± 0.57). During a six-week rehabilitation patients participated in a structured treatment and teaching program (STTP). Following the inpatient treatment the children and adolescents were monitored over a period of 24 months (physical examination, measurements of BMI, BMI-SDS, body composition, carotid intima-media thickness, laboratory parameters, blood pressure, and standardized questionnaires to assess socio-demographic, socio-economic parameters, eating behavior, well-being, quality of life, intelligence, intrafamilial conflicts, self-efficacy, resilience, sense of coherence, stress-management, social support, and actual body shape). *Results*: 66% of the children and adolescents showed non-normal laboratory parameters as well as higher blood pressure and/or an increased carotid intima-media thickness. Mean thickness of carotid intima-media was 0.53 ± 0.09 mm (range, 0.40–0.80); 15% of the patients showed a normal range (<0.45 mm), 40% slightly elevated (0.45–0.50 mm) and 45% an elevated (>0.50 mm) thickness. After an inpatient treatment lasting 40.4 ± 4.1 (range, 28–49) days, children and adolescents reached a mean weight reduction of 5.52 ± 3.94 (0.4–13.3) kg (*p* < 0.01) accompanied by a reduction of body fat mass. Performing multivariate analyses, the most important psychological factors associated with long-term weight reduction were identified (R-square = 0.53): Well-being (β = −0.543), resilience (β = 0.434) and intrafamilial conflicts (β = 0.315). *Conclusion*: The different parameters (*i.e.*, resilience, intrafamilial conflicts, structured daily schedule) have demonstrated their utility and strategies should be developed allowing an adaption of these into the STTPs and the integration of intervention into the therapeutic setting.

## 1. Introduction

Worldwide, overweight and obesity are known as posing serious health risks. According to WHO, obesity is one of the most prevalent chronic disorders in children and adolescents [1]. Up to 40% of overweight children and up to 80% of overweight adolescents will also be overweight or obese during adulthood [2]. Being overweight and obese is accompanied by various comorbidities. These include arterial hypertension, metabolic disorders such as dyslipidemia, diabetes mellitus, and high inflammatory activity as well as a reduced quality of life and well-being [3,4,5,6,7,8,9,10,11,12,13,14].

Understanding the complex etiology and developing successful methods of prevention and therapy for overweight and obesity have remained elusive. Most studies published to date demonstrate either low or only short-term positive effects [15,16,17,18,19]. Most frequently, interventions work only for the short-term; patients have difficulty in maintaining the skills and lifestyle modifications they have acquired for continuing weight reduction, and stabilizing body weight over longer periods of time. These problems raise questions about predictors and determinants for the development of overweight and obesity and their role in initial weight loss during an inpatient treatment program in the context of a medical rehabilitation. Hence, it was the aim of the present long-term trial to identify such parameters and to evaluate their long-term effects.

## 2. Patients and Methods

The present prospective, multicenter trial was performed in cooperation with the following hospitals specialized in rehabilitation and the treatment of children and adolescents: MEDIGREIF-Inselklinik Heringsdorf GmbH, Ostseebad Heringsdorf, Ostsee-Kurklinik Fischland GmbH, Ostseebad Wustrow, Fachklinik Sylt für Kinder und Jugendliche, Westerland, and Ostseestrandklinik Klaus Störtebeker, Kölpinsee, all in Germany. Totally, 167 Caucasian children and adolescents with overweight (BMI (body mass index)/BMI-SDS (body mass index standard deviation score) > 97 percentile [20]) and obesity (BMI/BMI-SDS > 99 percentile [20]) who were successively admitted to participate in a structured treatment and teaching program (STTP) for weight reduction [20,21] in one of the hospitals mentioned above, were screened to include in the trial. Hence, 159/167 (95%) fulfilled the inclusion criteria and were included in the trial. During the study period, 16 children and adolescents (10%) were excluded. Reasons for exclusion were problems of treatment adherence (cognitive inability to internalize the principles of eating and sports to guarantee weight reduction) in 5 persons and loss to follow-up in 11 patients. In total, 143/159 children and adolescents (90%) completed the trial.

The STTP [20,21] consisted of 28 therapeutic sessions (most important topics addressed: psychological and psychosocial backgrounds, nutrition and healthy cooking, physical activity and sports, medical impacts of overweight and obesity, leisure time management, and training of dealing with modern technologies (computers, TV, mobile phones, *etc.*)) with a duration of 45 min each. At the beginning of the trial (t0); after 6 weeks rehabilitation during inpatient treatment (t1); after 3 (t2), 6 (t3), 9 (t4), and 12 months (t5); and at the end of the follow-up period, 24 months after hospital demission (t6), the following examinations were performed:

1. In all patients, physical examinations were performed (t0 and t1).
2. Measurements of height and weight were assessed with patients wearing light clothing and without shoes. BMI and BMI-SDS were calculated according to the formulae “BMI = kg/m^2^” and “BMI-SDS = ([BMI/M_(t)_]L_(t)_ − 1)/(L_(t)_ × S_(t)_)” (M_(t)_, L_(t)_ and S_(t)_ are pre-defined parameters depending on age_(t)_ and sex [20]) (t0, t1, t2, t3, t4, t5, and t6).
3. Body composition analyses were done using a Body composition analyzer (BC418MA, TANITA Europe GmbH, Sindelfingen, Germany) (t0 and t1).
4. Quantitative B-mode ultrasound (Toshiba, Type SSA-350A “Corevision PRO”, 8 MHz, Linear Sonde Type PLF-805 St, Toshiba Medical Systems, Neuss, Germany) measurement of carotid intima-media thickness (IMT) were done by one physician performing 5 measurements on each side and calculating the mean (t0). Definition of age-adjusted normal values was according to the German standard [22].
5. Laboratory parameters (TSH (chemiluminescence-assay), total cholesterol (enzymatically), LDL-cholesterol (enzymatically), triglyzerides (enzymatically), uric acid (enzymatically), C-reactive protein (CRP) (turbidimetry), fasting blood glucose (enzymatically) and glucose values (enzymatically)) following an oral glucose tolerance test (75 g glucose, oGTT [23]) (t0).
6. Blood pressure in the sitting position was measured after the patients had rested for 10 min by using a standard sphygmomanometer according to the World Health Organization (WHO) recommendations [24]. In all patients, a 24-h-monitoring was performed (Premo Trend, Zimmer Elektromedizin, Neu-Ulm, Germany) (t0).
7. All patients completed standardized questionnaires to assess socio-demographic and socio-economic parameters (family history and status; social status; education; profession of parents; and time spent using a computer, watching TV, and playing sports), eating behavior (kind and amounts of food and liquids in respect of mean main meals), well-being, quality of life (disease-related and weight-related), motivation (intrinsic and extrinsic), intelligence, intrafamilial conflicts (*i.e.*, conflicts with parents and siblings), self-efficacy, resilience, sense of coherence, stress-management, social support and actual body shape (Table 1) (t0, t1, t6).

**Table 1:** Psychological questionnaires used in the present trial (* reported from the literature).

Variable	Questionnaire	Cronbach‘s Alpha *
Well-being	Well-being questionnaire (Berner Fragebogen zum Wohlbefinden (BFW)) [25]	0.84
Quality of life	Questionnaire for the assessment of disease-related quality of life (Fragebogen zur Erfassung der gesundheitsbezogenen Lebensqualität von Kindern und Jugendlichen (Kindl-R)) [26]	0.49–0.86
Weight-related quality of life	Questionnaire for the assessment of weight-related quality of life (Gewichtsbezogener Lebensqualitätsfragebogen (GW-LQ-KJ)) [27]	0.83
Motivation	Questionnaire for the assessment of intrinsic and extrinsic motivation [28]	-
Intelligence	Assessment of individual‘s intelligence (Wortschatztest, Zahlenfolgetest aus Grundintelligenztest Skala 2—CFT 20) [29]	-
Intrafamilial conflicts	Questionnaire for the assessment of intrafamilial conflicts (Familienklimaskalen (FKS)) [30]	0.60–0.73
Self-efficacy	Assessment of general self-efficacy (Allgemeine Selbstwirksamkeitserfahrung (SWE)) [31]	0.82
Resilience	Assessment of resilience (Resilienzskala (RS-11)) [32]	0.76
Sense of coherence	Children’s sense of coherence scale (CSOC) [33]	0.64
Stress-management	Questionnaire for the assessment of stress and its management (Fragebogen zur Erhebung von Streßerleben und Streßbewältigung im Kindesalter (SSK)) [34]	0.77–0.92
Social support	Berliner social support Scale (BSSS) [35]	0.60–0.87
Actual body shape	Figures of gender-specific body shape	-

### 2.1. Ethics Vote

The trial was approved by the local Ethics committee (Ernst-Moritz-Arndt-University, Greifswald, Germany, BB 37/08, 06.06.2008).

### 2.2. Statistical Analysis

Statistical analyses were performed using the program SPSS 22.0 (Statistical Package for the Social Sciences®, IBM, Armnok, New York, NY, USA). Values showing normal distribution were registered as mean (MW) ± standard deviation (SD), non-normal distributed values were given as median and range. Correlations were calculated according to Pearson and multivariate analyses (stepwise and ANOVA, respectively) were performed. Comparisons were evaluated with chi-square-tests or Fisher’s exact test in case of *n* < 6. Paired Student’s *t*-test and Wilcoxon-tests were used to compare the mean values. Significance was set at *p* < 0.05. Two-tailed significance tests were used throughout.

## 3. Results

### 3.1. Baseline Characteristics (t0)

Characteristics of the patients at baseline are shown in Table 2 and Table 3. 66% of the children and adolescents showed non-normal laboratory parameters as well as higher blood pressure and/or an increased carotid intima-media thickness. None of the patients suffered from arterial hypertension. Mean thickness of carotid intima-media was 0.53 ± 0.09 mm (range, 0.40–0.80); 15% of the patients showed a normal range (<0.45 mm), 40% slightly elevated (0.45–0.50 mm) and 45% an elevated (>0.50 mm) thickness.

After an inpatient treatment lasting 40.4 ± 4.1 (range, 28–49) days, children and adolescents reached a mean weight reduction of 5.52 ± 3.94 (0.4–13.3) kg (*p* < 0.01) accompanied by a reduction of body fat mass (Table 4). The mean weight reduction during the trial corresponds to a reduction by about 6.5% of the body weight at baseline. There were no differences between patients with overweight (BMI/BMI-SDS ≤ 99 percentile) and obesity (BMI/BMI-SDS > 99 percentile) or between males and females.

**Figure 1:**
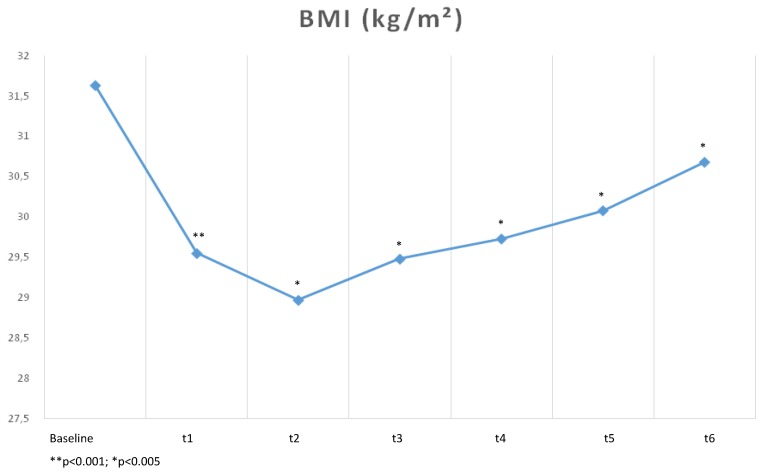
Changes of BMI during the trial.

**Figure 2:**
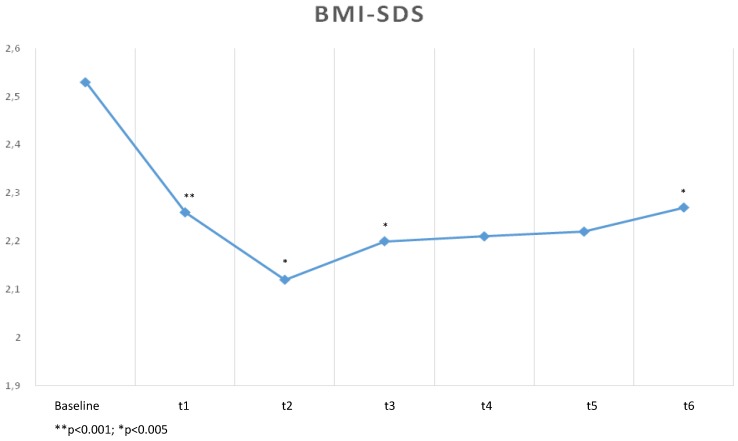
Changes of BMI-SDS during the trial.

Until the end of the trial (t6), BMI and BMI-SDS showed a slight increase, but both were significantly lower in comparison to baseline (t0) (Figure 1 and Figure 2).

**Table 2:** Baseline-characteristics (physical examination, laboratory parameters, blood pressure) (t0) of the 143 patients included in the trial and completed it. * median.

Parameter	MW ± SD	Min.	Max.
Number (*n*)	143	-	-
Age (years)	13.9 ± 2.4	9.3	18.4
Females (%)	62	-	-
Height (m)	1.62 ± 0.12	1.30	1.97
Weight (kg)	84.1 ± 22.6	40.8	155.2
BMI (kg/m^2^)	31.2 ± 5.4	20.3	51.4
BMI-SDS	2.51 ± 0.57	0.6	4.0
Obesity (%)	56	-	-
Duration of inhouse treatment (days)	40.4 ± 4.1	28	49
Fasting blood-glucose (mmol/L)	4.17 ± 0.5	2.0	5.1
oGTT: Blood-glucose 2 h after glucose-loading (mmol/L)	5.2 ± 0.8	3.0	6.9
Pathological oGTT (%)	0	-	-
Total cholesterol (mmol/L)	4.5 ± 0.9	2.6	7.4
Total cholesterol ≥ 5.2 mmol/L (%)	25	-	-
LDL-cholesterol (mmol/L)	2.8 ± 0.8	1.2	5.8
LDL-cholesterol ≥ 2.6 mmol/L (%)	73	-	-
HDL-cholesterol (mmol/L)	1.6 ± 0.3	0.9	2.6
HDL-cholesterol < 1.0 mmol/L (%)	5	-	-
Triglycerides (mmol/L)	1.1 ± 0.5	0.4	2.9
Triglycerides ≥ 1.70 mmol/L (%)	18	-	-
TSH (μIU/mL)	2.9 ± 1.3	0.2	7.8
Hypothyreosis (TSH > 4.00 μIU/mL) (%)	21	-	-
Uric acid (μmol/L)	359.8 ± 83.3	191.0	631.0
Hyperuricaemia (≥ 440 μmol/L) (%)	24	-	-
CRP (mg/dL)	0.5 *	0.5	4.0
CRP > 0.5 mg/dL (%)	34	-	-
Systolic blood pressure (mmHg)	121.7±9.2	99	150
Systolic blood pressure > 140 mmHg (%)	4	-	-
Diastolic blood pressure (mmHg)	68.3 ± 6.5	55	84
Diastolic blood pressure > 80 mmHg (%)	2	-	-
24-h-blood pressure systolic (mmHg)	119.0 ± 9.4	95	150
24-h-blood pressure diastolic (mmHg)	65.9 ± 6.5	51	81
Systolic day-/night-difference (mmHg)	16.1 ± 9.1	2	35
Diastolic day-/night-difference (mmHg)	11.5 ± 7.6	0	24

**Table 3:** Socio-demographic data of the 143 patients included in the trial and completed it at baseline (t0).

Parameter	Number (*n*)	Percentage (%)
Father		
Educational level		
High	19	13
Medium	70	49
Low	33	23
Unknown	21	15
Body weight		
Normal	70	49
Overweight/obese	73	51
Mother		
Educational level		
High	19	13
Medium	90	63
Low	26	18
Unknown	8	6
Body weight		
Normal	43	30
Overweight/obese	100	70

Changes of body weight, BMI and body composition.

**Table 4:** Changes of weight, BMI, BMI-SDS and body composition during the period of inpatient treatment (baseline (t0) *vs.* at the end of inpatient treatment (t1)) in 143 children and adolescents.

Parameter	Baseline (t0)			At the End of Inpatient Treatment (t1)			*p*-Value
	MW ± SD	Min.	Max.	MW ± SD	Min.	Max.	
Weight (kg)	84.1 ± 22.6	41	155	78.7 ± 20.5	38	144	<0.01
BMI (kg/m^2^)	31.2 ± 5.4	20	51,4	29.3 ± 4.9	19	47	<0.01
BMI-SDS	2.51 ± 0.57	0.6	4,0	2.25 ± 0.57	0.13	3.8	<0.01
*Body composition*							
Percentage of body fat (%)	38.6 ± 6.5	24	53	35.2 ± 6.6	19.4	50.8	<0.01
Fat mass (kg)	34.2 ± 12.6	12	82	29.2 ± 10.3	10.3	61.2	<0.01
Fat-free mass (kg)	52.4 ± 12.9	29	90	51.8 ± 13.2	25.6	89.3	<0.01

### 3.2. Socio-Psychological Parameters

Ninety-one percent of the children and adolescents reported an intrinsic motivation with regard to participation in the STTP for weight reduction. Prior to hospital admission, the patients spent 2.4 ± 1.5 h/day watching TV and 1.7 ± 1.4 h using a computer, while, at the end of the follow-up period, children and adolescents reported to spend 1.9 ± 3.4 h/day watching TV and 1.4 ± 2.1 h using a computer (*p* = 0.64). Those patients who spent more time with the computer and/or TV revealed a weaker reduction of BMI-SDS.

At baseline, only 6% of the patients were engaged in sport activities of ≥4 h or more per week.

The results of correlation analyses are shown in Table 5: Children and adolescents reporting low caloric intake at baseline reached a lower reduction of BMI-SDS by the end of the trial. It was also observed that a stronger reduction of BMI-SDS 6, 9 and 12 months after leaving the hospital correlated to an improved reduction by the end of the trial period. The results were similar to results regarding a structured daily schedule, as well as higher personal resilience.

**Table 5:** Results of correlation analyses (target parameter: reduction of BMI-SDS by the end of the trial [t6]).

Parameter	Correlation Coefficient (*r*)	*p*-Value
Reduction of BMI-SDS 6 months after inpatient treatment (t3)	0.25	0.018
Reduction of BMI-SDS 9 months after inpatient treatment (t4)	0.39	<0.001
Reduction of BMI-SDS 12 months after inpatient treatment (t5)	0.52	<0.001
Breakfast—low caloric intake at baseline (t0)	−0.23	0.036
Breakfast—medium caloric intake at baseline (t0)	−0.24	0.025
Lunch–low caloric intake at baseline (t0)	−0.29	0.007
Lunch–medium caloric intake at baseline (t0)	−0.23	0.030
Dinner–low caloric intake at baseline (t0)	−0.26	0.017
Dinner–medium caloric intake at baseline (t0)	−0.23	0.035
Low daily caloric intake at baseline (t0)	−0.26	0.013
Medium daily caloric intake at baseline (t0)	−0.32	0.003
Structured daily schedule at baseline (t0)	0.26	0.015
Time spending with computer/TV per day at baseline (t0)	−0.27	0.028
Resilience at baseline (t0)	0.24	0.024

Performing multivariate analyses, the following psychological factors significantly associated with long-term weight reduction were identified (R-square = 0.53): well-being (t6) (β = −0.543), resilience (t0) (β = 0.434) and intrafamilial conflicts (t6) (β = 0.315) (Table 6).

**Table 6:** Results of multivariate analyses.

Parameter	β	T	*p*-Value
Daily caloric intake (t0)	0.24	2.72	0.008
Intrafamilial conflicts (t6)	0.32	2.59	0.012
Well-being (t6)	−0.54	−4.70	<0.001
Resilience (t0)	0.43	3.67	<0.001
BMI at the end of inpatient treatment (t1)	0.25	2.88	0.005
Caloric intake at breakfast (t0)	0.25	2.86	0.006
Stress management (t6)	0.24	2.60	0.011
Duration of overweight/obesity (t0)	0.19	2.25	0.027
Mother‘s profession (t0) *	0.18	2.07	0.043

* profession was scaled: 0 (very low)–6 (very high), based on monthly income according to German standards [36].

All other parameters included in the models (sex, age, BMI-SDS, socio-demographic and socio-economic parameters (with the exception of mother’s profession), eating behavior (with the exception of the caloric intake at breakfast) and other psychological factors) revealed no correlations or associations.

## 4. Discussion

The aim of the trial was to uncover potential medical, socio-economic and psychological predictors which play a significant role in successful weight loss in overweight and obese children and adolescents during a STTP [20,21] in an inpatient rehabilitation setting; the results would be of significant interest not only for patients but also for physicians, psychologists and the national health systems. During the last decades, a substantial increase in prevalence of overweight and obesity worldwide has been documented [37,38,39,40,41]. Early and effective therapeutic strategies are mandatory to successfully confront this tendency and to prevent the development of premature co-morbidities and the high rates of early mortality.

In the present trial, approximately two-thirds of the children and adolescents had a higher intima-media thickness, elevated blood pressure levels and/or higher concentrations of lipids and CRP; all together, these are risk indicators for metabolic and cardiovascular disorders. These findings are in agreement with numerous previous studies [42,43,44,45,46].

Weight reduction which followed the participation in this STTP [20,21] performed during a six-week inpatient rehabilitation, was highly effective over a period of 24 months. Although best weight reductions were reached three and six months after participation in the STTP [20,21], the mean weight at the end of the trial was still significantly lower than body weight at baseline. Hence, the present trial showed a more effective weight reduction than previous trials [17,47,48,49]. Reasons for this positive tendency may be the small modifications [21] to the original STTP [20] and the inpatient treatment during a rehabilitation in specialized hospitals over periods of six weeks. However, the authors of this article also recognize that inpatient treatment over such a long period of time is not suitable for all children and adolescents with overweight/obesity. Further trials have to deal with the use of such a STTP [20,21] in ambulatory settings too.

As most important predictors for effective long-term weight reduction, the present trial identified: A good weight reduction already in progress during the participation in the STTP [20,21], low BMI at the time of hospital demission after finishing the inpatient rehabilitation, a low caloric intake at breakfast, low daily caloric intake, a low level of intrafamilial conflicts, high personal well-being, good resilience, good stress management, a shorter duration of being overweight and obese, and higher education and profession of the patients’ mothers. In most aspects, these findings are congruent to the data reported in the literature: Kurt and Schaffrath [49] and Benecke and Vogel [50] found in their studies a higher risk for the development of overweight and obesity in families with lower educational levels and when parents are not well educated and have lower ranked professions. Reasons for this association be possibly due to differences in self-perception, an inferior diet, less physical activity and frequently a non-structured daily schedule. Moreover, children and adolescents from lower social classes spend more time in front of TV and computer [49]. With regard to psychological aspects: Patients’ well-being, resilience, level of intrafamilial conflicts and stress management seem to be very important for long-term weight control. An improvement of a patient’s resilience is possibly associated with a better compensation of internal and external stress factors. In particular stress factors are often responsible for low physical adherence, poor medical prevention, eating disorders or a high caloric intake [51,52,53,54,55], and similarly for intrafamilial conflicts. In families with higher rates of conflicts and poor intrafamilial cohesion children and adolescents have an increased risk for the development of behavioral patterns, depression, other psychological disorders, but also overweight and obesity [56,57,58]. In contrast, healthy family environment and cohesion are protective with regard to physical and mental health and are sources of high resilience [57,59,60]. Another important factor for weight reduction and the maintenance of normal body weight seems to be a structured daily schedule. In Germany, in 2014, a nationwide trial sponsored by one of the largest health care insurance companies (Allgemeine Ortskrankenkasse (AOK)) was published. In this study it was clearly demonstrated that a structured daily schedule with meals at specified times taken regularly with other members of the family is associated with a lower incidence of overweight and obesity [61]. Similar results were reported by Nicklas *et al.* in 2004 [62].

Interestingly, the level of motivation in the patient did not play a central role or guarantee long-term weight reduction after participation in a STTP [20,21]. This finding in some ways contradicts previous trials. However, this finding has perhaps a bias due to the design of the present trial. The IDA-Insel study was not designed as population-based, but was aimed specifically for children and adolescents participating in a STTP [20,21] during an inpatient rehabilitation. Baseline characteristics show that more than 90% of the patients participated in the therapy due to their own motivation. At a minimum, these 90% had a very high intrinsic motivation for weight reduction. Hence, the cohort investigated is in some way a positive selection of patients. It remains an open question whether the results were comparable in cohorts with lower intrinsic or mainly extrinsic motivation.

## 5. Conclusions

Predictors and determinants associated with long-term weight reduction and identified in the present trial are very important in the context of established therapeutic settings. The different parameters (*i.e.*, resilience, intrafamilial conflicts, and structured daily schedule) have demonstrated their utility and strategies should be developed allowing an adaption of these into the STTPs [20,21] and the integration of intervention into the therapeutic setting. In particular, in the future, STTPs should focus much more on strategies for the improvement of resilience, stress management, prevention of intrafamilial conflicts and heightened personal well-being. Moreover, caloric intake and a structured daily schedule should be part of the strategy in a more intensive way. However, future trials are required to understand how to incorporate such parameters best and to evaluate the effects of these interventions.
